# American Medical Association

**Published:** 1884-06-20

**Authors:** 


### AMERICAN MEDICAL ASSOCIATION.

It now appears that there were 1,300 doctors present at the late meeting of the American Medical Association at Washington City. Among these it is stated, that there were comparatively very few representatives from the Southern States. And yet the South was honored with a President-elect for the ensuing year in the person of Dr. H. T. Camphell, of Augusta.

The papers read before the Secions are spoken of as not presenting anything especiall new. Indeed it may be truthfully said in the language of the Va. Med. Monthly, that: "One of the chiefest things that many doctors go to assemblages of such kind as this National Association for, is that they may run for office to lobby and bargain. Have medical bodies come down to such a level? Why is it that the last day is not attended as well as the first or second day? A clique is at hand; a caucus organized; a new mhasure set afoot to do what? That friends may be carried to the front—and the science and art of professional study is sometimes left too far in the background. If such a failure of profitable result comes of organization, then societies ought to be abolished, and individual effort must be resumed."

We are pleased to mention an interesting little incident in the closing exercises of the Association which speaks well for the dignity and moral tone of the body:

A member introduced a resolution which stated "That as many members of the Asssciation were infidels, free thinkers, etc., the custom of opening the sessions of the annual meeting with prayer was an imposition, and that therefore it be abolished."

A motion to lay on the table was promptly made, and as promptly carried without a single dissenting vote. Perhaps the mover of this resolution had diligently watched and waited to do something and to figure in print among the great lights of the Association, and thus he did it!

